# Efficacy and Safety of *Xue-Fu-Zhu-Yu* Decoction for Patients with Coronary Heart Disease: A Systematic Review and Meta-Analysis

**DOI:** 10.1155/2021/9931826

**Published:** 2021-09-29

**Authors:** Shuo Zhang, Zhen-Lin Chen, Yu-Ping Tang, Jin-Long Duan, Kui-Wu Yao

**Affiliations:** ^1^School of Clinical Medicine (Guang'anmen Hospital), Beijing University of Chinese Medicine, Beijing, China; ^2^Guang'anmen Hospital, China Academy of Chinese Medical Sciences, Beijing, China; ^3^International Programs Office, Shaanxi University of Chinese Medicine, Xi'an, Shaanxi, China; ^4^Key Laboratory of Shaanxi Administration of Traditional Chinese Medicine for TCM Compatibility, Shaanxi University of Chinese Medicine, Xi'an, Shaanxi, China

## Abstract

**Objective:**

To systematically evaluate the efficacy and safety of XFZYD for coronary heart disease (CHD).

**Methods:**

A comprehensive literature search of randomized controlled trials using XFZYD for CHD was conducted in 10 electronic databases from their establishment to December 20, 2020. The researchers screened the relevant trials in NoteExpress, extracted the data in duplicate independently, assessed the risk of bias in the trials using the Cochrane collaboration tool, and then used Rev Man 5.3 for data analysis.

**Results:**

30 trials with 3126 participants were included for meta-analysis. The results showed that the clinical effects of XFZYD and its combination with chemical drugs (CD) were 1.13 (RR; 1.13; 95% CI, 1.03 to 1.24) and 1.26 (RR; 1.26; 95% CI, 1.20 to 1.32) times those of CD, respectively. And, it could also improve electrocardiogram effect, which was 1.63 (RR; 1.63; 95% CI, 1.04 to 2.53) times that of CD. XFZYD could not only decrease duration of angina pectoris and improve vascular endothelial function but also obviously reduce the TCM syndrome score. When used in combination with CD, it could also lower AF, correct the dyslipidemia, and reduce the blood viscosity.

**Conclusion:**

These results demonstrated that XFZYD had great advantages in treating CHD with no obvious adverse reactions. Therefore, it is believed that XFZYD is more suitable for CHD patients with clinical indicators of dyslipidemia, high blood viscosity, or vascular endothelial dysfunction. This study is the first systematic review and meta-analysis with some unique ways, including its comprehensiveness, large-scale search, the novelty of findings, and transparent approach.

## 1. Introduction

Coronary heart disease (CHD) is a heart disease caused by atherosclerotic lesions in the coronary arteries, which causes stenosis or obstruction of the vascular lumen, resulting in myocardial ischemia, hypoxia, or necrosis [1], and it is the main cause of death worldwide. Due to its high morbidity and mortality, CHD has gradually become an important public health problem worldwide, and it has attracted extensive attention at home and abroad. Standardized treatments include drug and revascularization therapy. Although some drugs can delay the development of CHD to a certain extent, long-term medication will cause adverse reactions; for example, depression is a side effect of medication, which may aggravate the development of CHD [2].

Traditional Chinese medicine (TCM) has long-term practice in the treatment of CHD, which belongs to the category of “chest pain” and “heartache” in TCM. Its onset is mostly caused by cold pathogen invasion, eating disorder, emotional disorder, weary internal injuries, and aging physical weakness, which leads to abnormal circulation of blood and Qi, and its pathogenesis is blockage of the cardiac vessels. *Xue-Fu-Zhu-Yu* decoction (XFZYD) has the effects of promoting blood circulation for removing blood stasis and regulating qi-flowing for relieving pain, which is a classic TCM formula for the treatment of blood stasis syndrome [3] and is widely used in a variety of cardiovascular diseases [4]. XFZYD is composed by 11 kinds of herbs, including Persicae Semen (Taoren), Carthami Flos (Honghua), Aurantii Fructus (Zhiqiao), Radix Platycodonis (Jiegeng), Medicinal cyathula root (Chuuanniuxi), Bupleuri Radix (Chaihu), Rehmanniae Radix (Shengdi), Angelicae Sinensis Radix (Danggui), Chuanxiong Rhizoma (Chuanxiong), Paeoniae Radix Rubra (Chishao), and Glycyrrhizae Radix Et Rhizoma (Gancao). Although there were many randomized controlled trials in the clinic to study the efficacy of XFZYD in the treatment of CHD, the research was fragmented and lacks systematization. Moreover, due to different sample sizes, usage and dosage, and outcome indicators, the evaluation criteria were not unified, and the research conclusions were not consistent. In addition, although there were several meta-analyses on the treatment of CHD with XFZYD currently [5–7], they only focused on one aspect of angina and did not include other symptoms or clinical classifications of CHD. Therefore, the comprehensiveness of XFZYD in the treatment of CHD cannot be obtained. In conclusion, this study aimed to comprehensively evaluate the efficacy and safety of XFZYD for CHD through systematic reviews and meta-analysis, and to summarize its potential therapeutic mechanisms, which was the first most large-scale and extensive research in this field, and the results will provide more effective and credible evidence for further guiding clinical practice and fill the knowledge gap of XFZYD for precise medication of CHD.

## 2. Data and Methods

This study was conducted and reported in accordance with the Preferred Reporting Items for Systematic Reviews and Meta-analyses (PRISMA) with Cochrane methodology [8]. This study has been registered, and the PROSPERO number is CRD42021229958.

### 2.1. Literature Search

From the establishment of each electronic database to December 20, 2020, randomized controlled trials that evaluated the effect of XFZYD for treating CHD were searched in the following 10 electronic databases: Cochrane Library (from 1966 to 2020), Medline (from 1946 to 2020), PubMed (from 1959 to 2020), Springerlink (from 1996 to 2020), Web of Science (from 1986 to 2020), ClinicalTrials.gov, Chinese Biomedical Literature Database (CBM, from 1978 to 2020), the Chinese National Knowledge Infrastructure (CNKI, from 1980 to 2020), Wanfang Database (from 1998 to 2020), Weipu Database (from 1989 to 2020), and Chinese Biomedical Literature Database (CBM, from 1978 to 2020). Forward and backward citation searching was conducted for all eligible trials. The following terms were used for searching: (“coronary heart disease” OR “xiongbi” OR “xintong”) AND (“xuefuzhuyu decoction” OR “xuefu zhuyu decoction” OR “xue fu zhu yu decoction” OR “xuefuzhuyutang” OR “xuefuzhuyu tang” OR “xuefu zhuyu tang” OR “xue fu zhu yu tang”) AND (“clinical trial” OR “randomized controlled trial” OR “randomized controlled trial” OR “lin chuang yan jiu” OR “lin chuang shi yan”). The language and status of publications in our literature search were not be specified. And we manually searched bibliographies of included trials and related reviews for additional references.

### 2.2. Criteria for Literature Inclusion

#### 2.2.1. Type of Research

This study included randomized controlled trials of XFZYD in the treatment of CHD. Trials were excluded if (a) they were not random; (b) no control group was used; (c) XFZYD was not used in the experimental group; (d) they are combined with other drugs; (e) experimental design was not rigorous, or statistical methods were inappropriate in trials; (f) they had indeterminacy of measurement index outcome criterion; (g) trials on effective analysis data cannot be obtained; (h) they are reviews, conference paper, case reports, experience sharing, etc.; (i) they are animal experiments; (j) they are repeatedly published articles and plagiarized studies.

#### 2.2.2. Study Subjects

Patients who were not restricted by age, gender, or nationality with CHD were eligible for inclusion in this study.

#### 2.2.3. Intervention Measures

The intervention measures in the experimental group should be XFZYD (no herb added or subtracted) or combined with the treatment of control group. And the control group should be chemical drugs (CD).

#### 2.2.4. Outcomes

The primary outcome was defined as angina frequency (AF) and duration of angina pectoris (DAP). The secondary outcomes were angina, which included clinical effect; electrocardiogram effect; blood lipid, which included total cholesterol (TC), triglyceride (TG), low-density lipoprotein cholesterol (LDL-C), and high-density lipoprotein cholesterol (HDL-C); hemorheology index, which included whole blood viscosity (WBV), plasma viscosity (PV), and fibrinogen (FB); vascular endothelial function indicators, which included NO and endothelin (ET); TCM syndrome score, and adverse reaction.

### 2.3. Study Selection and Data Extraction

According to the inclusion and exclusion criteria mentioned above, two researchers who participated in calibration and training exercises before starting the screening processes independently screened the titles and abstracts of potential eligible trials, which were in duplicate, and then they retrieved independently and reviewed the full text of the possible trials in duplicate based on the inclusion and exclusion criteria and compared their results. The screening process was conducted in Note Express 3.2.0.

We conducted various forms of calibration exercises and pilots before the data extraction process began. Two researchers used standardized tables to independently extract data in duplicate from all eligible trials according to the inclusion and exclusion criteria mentioned above. In case of disagreement, they agreed through discussion or submitted it to a third party for evaluation. And before the screening process, the third party used a standardized screening form and performed calibration exercises.

1. The basic information of the study (author's name, title of the study, year of publication, country/region, and publication status)
2. Study characteristics (sample size, source of cases, age, diagnostic criteria, and inclusion and exclusion criteria)
3. Intervention and control measures (dosage form, dose, and duration)
4. Research methodology (random scheme generation, allocation hiding, blind method, incomplete result data, selective reporting, other biases, and loss of follow-up)
5. Outcome measures

### 2.4. Assessment of Literature Quality

The methodological quality of each included study was assessed independently by two reviewers according to the Cochrane collaboration tool. It comprised the following 7 aspects: random sequence generation, allocation concealment, blind method, incomplete result data, selective reporting, and other biases. The quality assessment results of each item can be divided into three grades: “low risk,” “high risk,” and “unclear.” The more rigorous the design and the higher the methodological quality of each RCT, the lower the risk coefficient. For example, according to the random control table, the random sequence generation is “low risk.” Furthermore, the RCT that used allocation concealment and blind methods without incomplete outcome data, selective reporting, or other biases was considered as “low risk”; otherwise, it will be considered as “high risk.” If none of the above 7 aspects was reported in the trial, the aspect was considered to be “unclear.” When necessary, the consensus on this issue was studied with the help of a third party.

### 2.5. Statistical Analysis

Data analysis was performed using Rev Man 5.3 software. Both the continuous and dichotomous outcomes were derived from the included trials without any conversion. The dichotomous outcomes were described by relative risk (RR) and 95% confidence interval (CI); in addition, mean difference (MD) and 95% CI were used to describe the effect value of the intergroup comparison. Heterogeneity was determined according to the results of *I*^2^ test. *I*^2^<50% indicated the low heterogeneity of interstudy, and the fixed effect model was adopted. Furthermore, the random effect model was adopted when *I*^2^>50% [9]. Random effect model was also used to generate direct and mixed treatment comparison estimates. Subgroup analysis was conducted according to whether the experimental group was combined with chemical medicine and the different treatment methods in the control group. Inverted funnel plots were used to determine publication bias when the number of included studies exceeded 10 in the meta-analysis [10].

## 3. Results

### 3.1. Results of Our Literature Search

**Figure 1:**
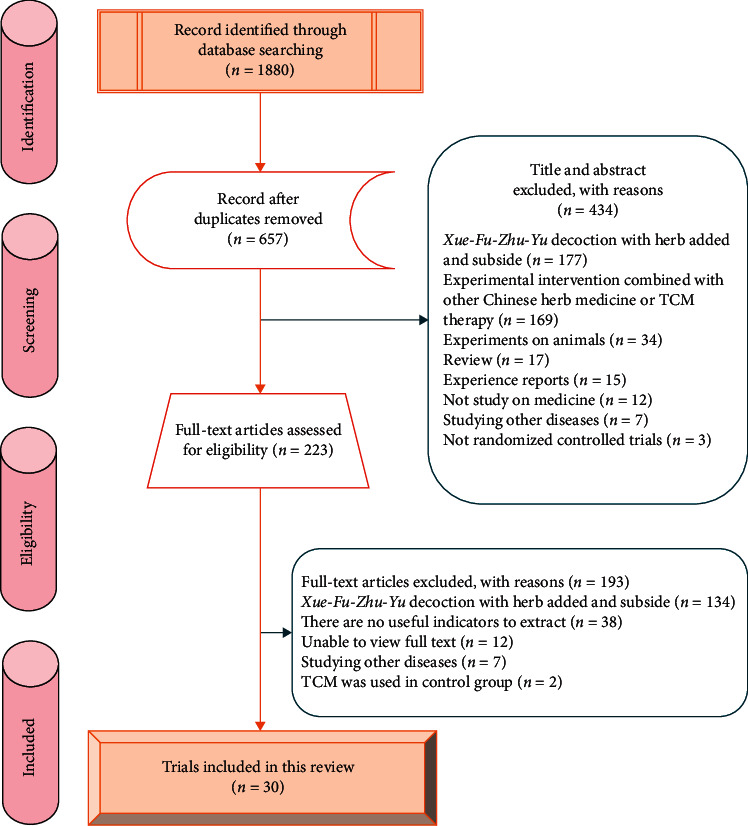
PRISMA flow diagram of literature selection.

Based on the above retrieval strategy, a total of 1880 potentially relevant trials were retrieved from 10 electronic databases, and 657 trials were retrieved after 1223 duplicates were deleted. After reviewing the titles and abstracts, 434 trials were excluded, because they did not comply with the inclusion criteria, and 223 trials initially met the predetermined requirements, and their full texts were read for detailed assessment. Finally, 30 trials were included for meta-analysis [11–40]. The PRISMA flow diagram of literature retrieval process is shown in Figure 1. All included trials have been published as full article.

### 3.2. Basic Characteristics of the Included Studies

Table 1 summarizes the basic characteristics of the eligible 30 trials and analyzed a total of 3126 patients with CHD. Sample sizes ranged from 40 to 300. In the included trials, XFZYD combined with chemical drug (XCWC) *vs.* chemical drug (CD) was used in 26 trials, and XFZYD *vs.* CD was used in 4 trials. In primary outcomes, 9 trials reported AF and 8 reported DAP. In terms of secondary outcome indicators, 22 trials reported clinical effect, 8 reported electrocardiogram effect, 9 reported TC, 6 reported TG, 8 reported LDL-C and HDL-C, 2 reported WBV, 3 reported PV and FB, 3 reported NO and ET, 4 reported TCM score, and 6 reported adverse effects. Duration of treatment was reported in all included trials from 14 days to 1 year.

### 3.3. Risk of Bias Assessment of the Literature Included in the Study

**Figure 2:**
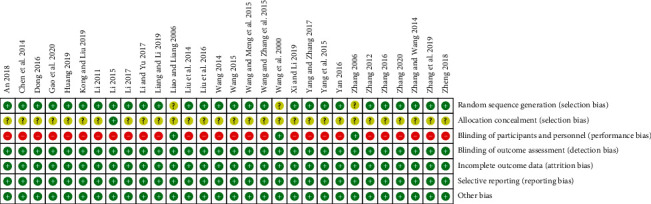
Methodological quality assessment results for all included trials.

**Figure 3:**
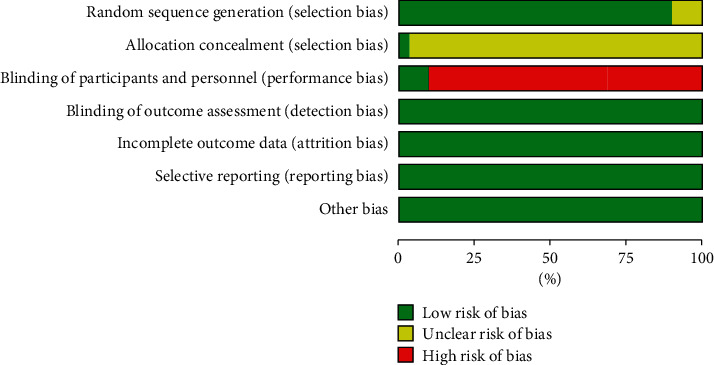
Percentage results of risk of bias for each of the included trials.

**Figure 4:**
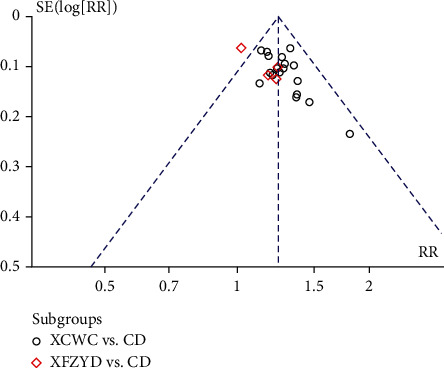
Inverted funnel plot of XFZYD in the treatment of CHD for clinical effect.

The methodological quality of 30 eligible trials is summarized in Figures 2–3, and the criteria in the Cochrane Handbook for Systematic Reviews of Interventions were used to assess the risk of bias in the study. Although randomization was announced in all of the included trials, 4 trials did not report the adequate sequence generation. 26 trials used random number table [11–29, 31–37], and 1 used block randomization [30]. 3 trials reported blind method [38–40], but only 1 trial reported allocation concealment [25].  Figure 4 provides the funnel plot of the trials for clinical effect, and Egger's test calculated *p* = 0.005, which revealed that there may be some publication bias in the trials.

### 3.4. Outcomes

#### 3.4.1. Angina (AF and DAP)

**Figure 5:**
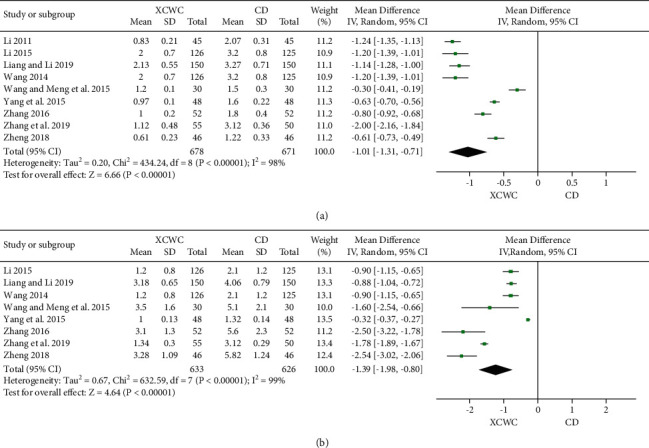
Forest plot of the trials showing angina in XCWC *vs.* CD: (a) AF; (b) DAP.

9 trials [15, 17, 19, 26, 27, 29, 31, 37] reported the AF before and after intervention. Compared with CD, XCWC exhibited significant lowering effects on AF (9 trials; *n* = 1349; MD, −1.01; 95% CI, −1.31 to −0.71; Figure 5(a)).

DAP was reported in 8 trials [15, 17, 19, 26, 27, 29, 31, 34], and the shorter the DAP, the less pain in patients. Meta-analysis evaluated a great lowering effect of XFZYD on DAP, and XFZYD could remarkably reduce DAP and relieve the pain of patients when compared with CD (8 trials; *n* = 1259; MD, −1.39; 95% CI, −1.98 to −0.80).

#### 3.4.2. Clinical Effect

**Figure 6:**
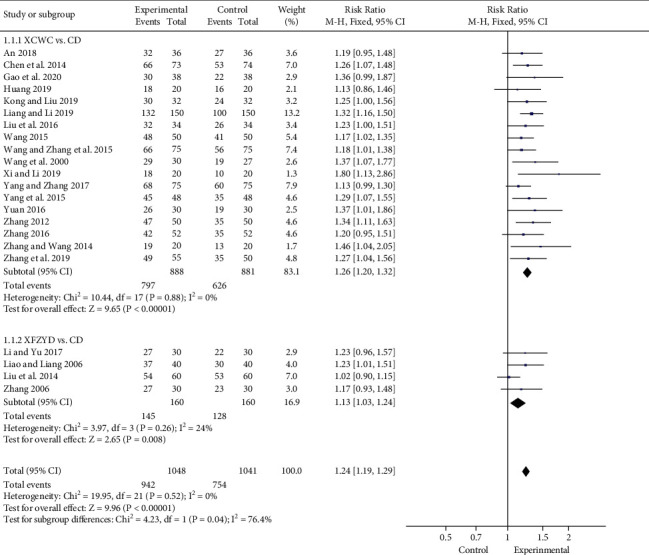
Forest plot of the trials showing clinical effect in different interventions.

The clinical effect was established with reference to the Standard of Diagnosis and Efficacy of Diseases in traditional Chinese Medicine, and the clinical effect standard was divided into cure, apparent efficacy, effectiveness, and ineffectiveness to evaluate the clinical effect of using XFZYD. The study treated patients with CHD, and 22 trials have been reported. In addition, meta-analysis revealed that XFZYD could significantly improve the clinical effect of CHD, and its effect was 1.24 times higher than that of CD (22 trials; *n* = 2089; RR; 1.24; 95% CI, 1.19 to 1.29; Figure 6). Among them, 18 trials [11, 13–18, 22, 24–26, 28, 30–32, 35, 36, 40] treated participants with XCWC and CD in experimental group and control group, respectively, and 4 trials [21, 33, 38, 39] used XFZYD and CD. Meta-analysis identified that XFZYD could observably enhance the clinical efficacy whether used alone or in combination with CD, which were 1.13 (4 trials; *n* = 320; RR; 1.13; 95% CI, 1.03 to 1.24) and 1.26 (18 trials; *n* = 1769; RR; 1.26; 95% CI, 1.20 to 1.32) times higher than those of CD, respectively.

#### 3.4.3. Electrocardiogram Effect

**Figure 7:**
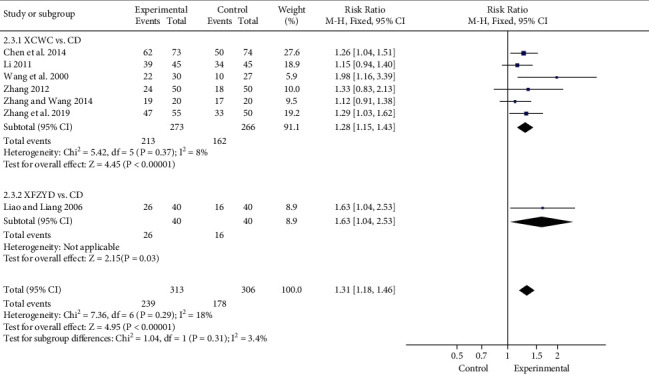
Forest plot of the trials showing electrocardiogram effect in different interventions.

The electrocardiogram effect was reported in 7 trials [17, 32, 35–38, 40], which was judged according to the changes of electrocardiogram before and after treatment. XFZYD showed an obvious improvement on electrocardiogram, and its effect was 1.31 times higher than that of CD (7 trials; *n* = 619; RR; 1.31; 95% CI, 1.18 to 1.46; Figure 7), which identified that XFZYD could significantly alleviate myocardial ischemia in patients. Additionally, the effect of XFZYD was 1.63 (1 trial; *n* = 679; RR; 1.63; 95% CI, 1.04 to 2.53) times higher than that of CD. Meta-analysis of 6 trials [17, 31, 35–37, 40] comparing XCWC with CD revealed a remarkable increasing effect of XCWC in electrocardiogram effect, which was 1.28 times higher than that of CD (6 trials; *n* = 539; RR; 1.28; 95% CI, 1.15 to 1.43).

#### 3.4.4. Blood Lipids (TC, TG, LDL-C, and HDL-C)

**Figure 8:**
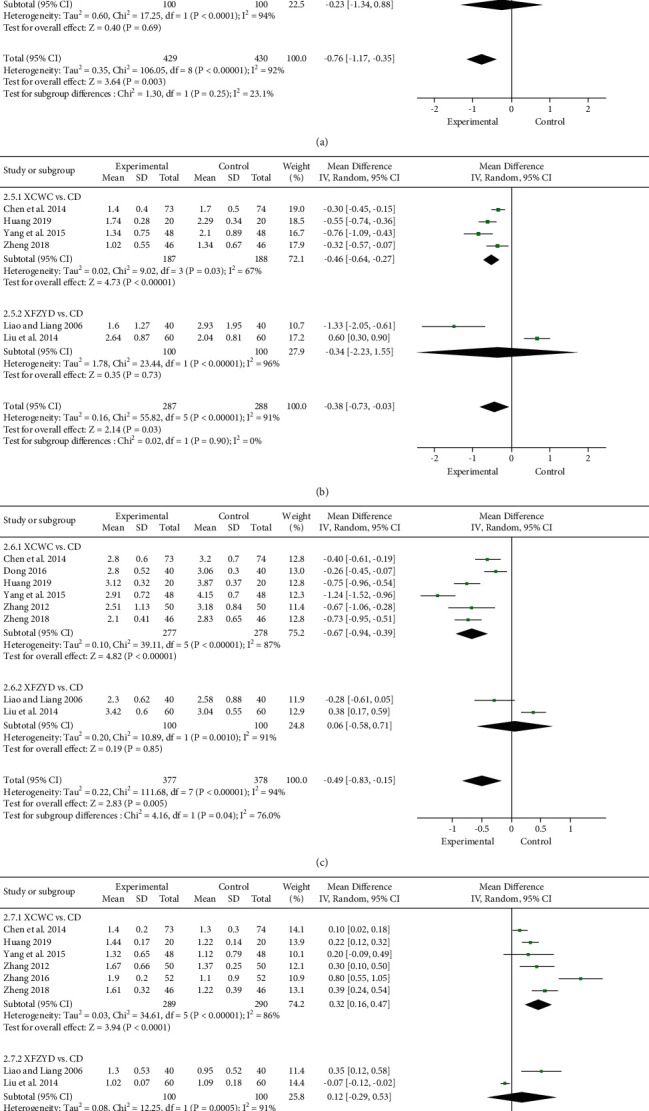
Forest plot of the trials showing blood lipids in different interventions: (a) TC; (b) TG; (c) LDL-C; (d) HDL-C.

The levels of TC and TG at baseline and after intervention were reported in 9 [13, 19, 23, 26, 31–33, 36, 37] and 6 [13, 19, 31–33, 37] trials, respectively, and the forest plots for the meta-analysis of each relevant drug are provided in Figures 8(a) and 8(b). Compared with CD, TC (7 trials; *n* = 659; MD, −0.91; 95% CI, −1.28 to −0.53) and TG (4 trials; *n* = 375; MD, −0.46; 95% CI, −0.64 to −0.27) were both significantly lower in the XCWC group. However, XFZYD did not show obvious advantages in TC (2 trials; *n* = 200; MD, −0.23; 95% CI, −1.34 to 0.88) and TG (2 trials; *n* = 200; MD, −0.34; 95% CI, −2.23 to 1.55).

Eight trials [13, 19, 23, 31–33, 36, 38] reported the levels of LDL-C at baseline and after treatment. The higher the LDL-C, the higher the risk of CHD. Compared with CD, LDL-C was remarkably lower in the XCWC group (6 trials; *n* = 555; MD, −0.67; 95% CI, −0.94 to −0.39; Figure 8(c)). However, there was no obvious difference between XFZYD and CD (2 trials; *n* = 200; MD, 0.06; 95% CI, -0.58 to 0.71).

HDL-C is a protective factor of CHD and can resist atherosclerosis, which was reported in 8 trials [13, 19, 26, 31–33, 36, 38]. In the meta-analysis, XCWC exhibited a significantly greater increase in HDL-C than that of CD alone (6 trials; *n* = 579; MD, 0.32; 95% CI, 0.16 to 0.47; Figure 8(d)), but XFZYD did not show remarkable advantages (2 trials; *n* = 200; MD, 0.12; 95% CI, -0.29 to 0.53).

#### 3.4.5. Hemorheology Index (WBV, PV, and FB)

**Figure 9:**
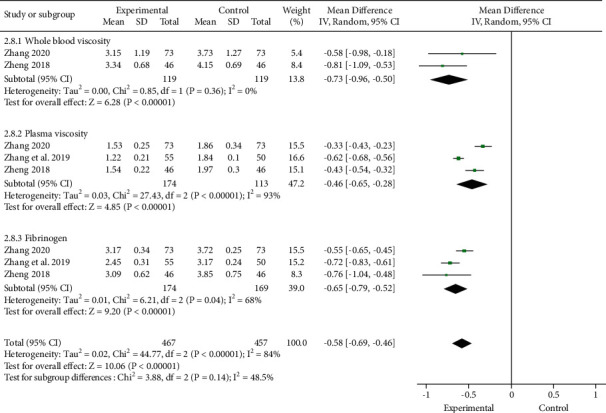
Forest plot of the trials showing hemorheology index.

Three trials [12, 17, 19] reported the level of hemorheology index at baseline and after intervention. Meta-analysis evaluated that the combination of XCWC and CD could significantly decrease hemorheology index to reduce blood viscosity (MD, −0.58; 95% CI, −0.69 to −0.46; Figure 9), which not only decreased WBV (2 trials; *n* = 238; MD, −0.73; 95% CI, −0.96 to −0.50) and PV (3 trials; *n* = 343; MD, −0.46; 95% CI, −0.65 to −0.28) but also exhibited a great advantage in reducing FB level (3 trials; *n* = 343; MD, −0.65; 95% CI, −0.79 to −0.52).

#### 3.4.6. Vascular Endothelial Function Indicators (NO and ET)

**Figure 10:**
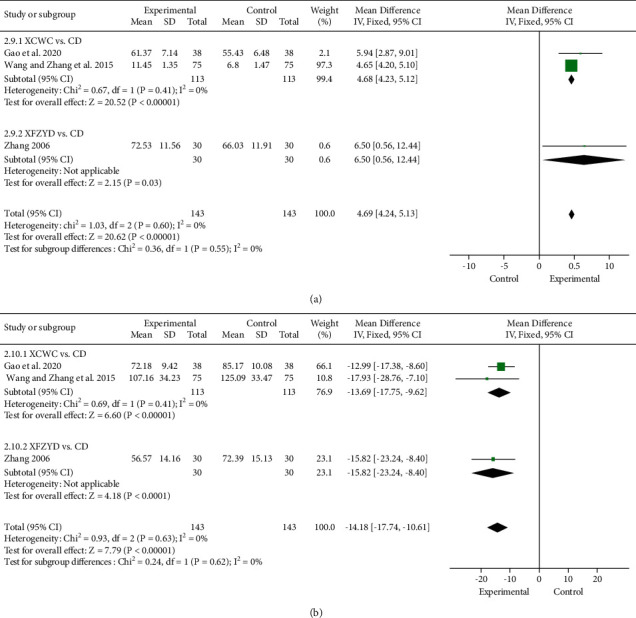
Forest plot of the trials showing vascular endothelial function indicators in different interventions: (a) NO; (b) ET.

Three trials [11, 30, 39] reported the levels of NO at baseline and after treatment. Compared with CD, NO level was remarkably increased in the XFZYD (1 trial; *n* = 60; MD, 6.50; 95% CI, 0.56 to 12.44; Figure 10(a)) and XCWC (2 trials; *n* = 226; MD, 4.68; 95% CI, 4.23 to 5.12; Figure 10(a)) group.

The effect of XFZYD on ET level was reported in the same trials as NO. Compared with CD, significant lowering effects on ET level by XFZYD alone (1 trial; *n* = 60; MD, −15.82; 95% CI, −23.24 to −8.4; Figure 10(b)) or in combination with CD (2 trials; *n* = 226; MD, −13.69; 95% CI, −17.75 to −9.62; Figure 10(b)) were both identified.

#### 3.4.7. TCM Syndrome Score

**Figure 11:**
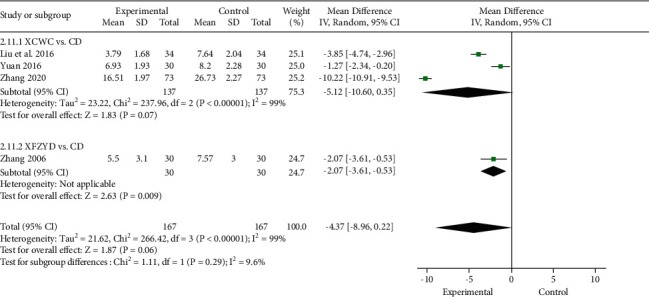
Forest plot of the trials showing TCM syndrome score in different interventions.

Four trials reported TCM syndrome score [12, 24, 25, 39]. The scoring method refers to the guiding principles for clinical research of new Chinese medicine [41], and scores are zero, one, two, and three points, respectively, according to the degree of clinical manifestations. The lower the score, the better the effect. Meta-analysis revealed that XFZYD could obviously reduce TCM syndrome score compared with CD (1 trial; *n* = 60; MD, −2.07; 95% CI, −3.61 to −0.53; Figure 11), but it did not show remarkable difference when combined with CD (3 trials; *n* = 274; MD, −5.12; 95% CI, −10.60 to 0.35).

#### 3.4.8. Adverse Reaction

**Figure 12:**
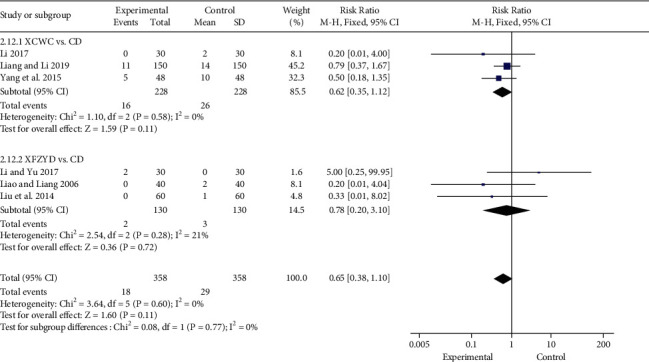
Forest plot of the trials showing adverse reaction in different interventions.

Six trials [15, 20, 21, 31, 33, 38] reported adverse reactions of the experimental group and control group after treatment, including headache, nausea, vomiting, diarrhea, extrasystole, hypotension, myocardial infarction, and elevated transaminase. However, when combined with CD, XFZYD alone (3 trials; *n* = 260; RR, 0.78; 95% CI, 0.20 to 3.10; Figure 12) or in combination with CD (3 trials; *n* = 456; RR, 0.62; 95% CI, 0.35 to 1.12; Figure 12) did not show obvious advantages.

## 4. Discussion

Among the 3 previous reviews, 2 were protocols [5, 6], and only 1 paper was the final study [7], which studied the efficacy of *Xue-Fu-Zhu-Yu* capsule in the treatment of unstable angina pectoris, and a total of 8 trials were included. It only had 4 outcomes including the incidence of a heart event, reduction of angina symptoms, ECG improvement, and quality of life. Our study was more optimized in terms of strict standards, rigorous screening process, multiple included trials, and comprehensive outcomes, and the results were more reliable, which provided help for clinical drug use from many aspects and filled the gap of precision medicine. In this study, both the number of included trials and the outcome measures were increased significantly. We comprehensively analyzed the efficacy and safety of XFZYD for CHD, explored the potential mechanism of XFZYD treatment from aspects of ECG improvement, blood lipid improvement, hemorheology, and vascular endothelial function, and then compared TCM syndrome scores of patients before and after treatment to judge the improvement of syndrome and life quality of patients. And the safety of XFZYD was evaluated according to the occurrence of adverse reactions after taking the drug.

The efficacy and safety of XFZYD for CHD were evaluated by meta-analysis on the basis of 30 trials and 3126 participants. The results showed that XFZYD alone or in combination with CD could significantly improve the clinical effect at 1.13 and 1.26 times higher than that of CD alone. At the same time, XFZYD could also obviously improve the curative effect of electrocardiogram and the condition of myocardial ischemia in patients, which was 1.63 times that of CD. Using XFZYD alone could not only reduce DAP, increase the level of NO, and lower the level of ET to improve vascular endothelial function, but also remarkably decrease TCM syndrome score and alleviate the symptoms of patients. Comparing with XFZYD alone, its combination with CD could not only reduce AF to relieve the pain of patients with CHD, decrease the levels of TC, TG, and LDL-C, and increase HDL-C, thereby correcting the dyslipidemia, but also lower the blood viscosity by decreasing the levels of WBC, PV, and FB. Therefore, XFZYD can treat CHD through multitarget comprehensive intervention. This study is the first systematic review and meta-analysis with some unique ways, including its comprehensiveness, large-scale search, the novelty of findings, and transparent approach, which provided a strong evidence support for the accurate use of medicine for CHD in clinics.

XFZYD can demonstrate its unique advantages in the treatment of CHD through the multitarget comprehensive action of multiple drugs. Taoren and Honghua promote blood circulation to arrest pain, Chishao and Chuanxiong promote blood circulation for removing blood stasis, Niuxi activates blood to promote menstruation and ensures proper downward flow of the blood, Shengdi and Danggui nourish Yin and blood, Jiegeng and Zhiqiao promote the circulation of Qi, Chaihu disperses stagnated hepatoqi, and Gancao coordinates the drug actions of a prescription. So, XFZYD can activate blood stasis and dissipate Qi, and then all symptoms can be cured, which is a good TCM formula for treating blood stasis. Modern pharmacological researches have shown that Taoren had antithrombotic, anticoagulant, and lipid-lowering effects, which could inhibit atherosclerotic plaque formation, prevent myocardial infarction, and improve hemodynamics [42]. Hydroxysafflor yellow A, one main bioactive component of Honghua, may inhibit coronary artery endothelial cell damage by increasing NO expression and release and hinder platelet aggregation, which could play an antiatherosclerotic effect [43]. The combination of Taoren and Honghua could not only significantly reduce blood viscosity through decreasing the concentration of FB and improving the aggregation of red blood cells and platelets, but also participate in various biological processes and signal pathways, as well as interfering with the occurrence and development of CHD, which played a protective and repairing effect on the cardiovascular system. Chuanxiong had the effects of antiatherosclerosis, lipid-lowering, dilating blood vessels, antiplatelet aggregation, antithrombosis, and so on [44]. The peony total glycosides of Chishao had the effects of antiatherosclerosis and stabilizing plaque through lipid-lowering, anti-inflammatory, and inhibiting angiogenesis [45], which could also obviously improve hemorheology, protect vascular endothelial function, and play a role in antiatherosclerosis; furthermore, it could improve myocardial ischemia when used with Chuanxiong [46]. Niuxi could not only reduce WBV and red blood cell aggregation index, but also prolong prothrombin time. Shengdi could lower blood lipids [47], protect the cardiovascular system, improve cell function, and avoid ischemic injury [48]. Danggui could decrease vascular resistance, reduce thrombosis, and improve blood circulation [49], thereby reducing blood lipids and atherosclerosis. Jiegeng could resist oxidation, lower blood lipids, and protect myocardium [50]. Naringin of Zhiqiao could not only reduce TC through multiple mechanisms, but also inhibit platelet aggregation [51], and hesperetin also had the effect of inhibiting platelet aggregation [52]. Chaihu had antioxidant, anti-inflammatory, and lipid-lowering effects, and it could regulate the blood coagulation state, which had a good effect on the treatment of cardiovascular diseases [53]. Gancao could significantly regulate lipid metabolism and play the purpose of treating atherosclerosis through antioxidation and anti-inflammatory effects [54].

XFZYD not only had anti-inflammatory, antioxidant, antiplatelet aggregation, and antitumor effects [55–57], but also could promote blood circulation, eliminate blood stasis, and make myocardial cells resistant to ischemic damage [58]. Furthermore, it could also improve microcirculation and vascular endothelial function and delay the formation of atherosclerosis [59]. Animal experiments found that XFZYD could not only reduce WBV and PV to improve hemorheology disorders, but also decrease TC, TG, and LDL-C and improve HDL-C [60]. And it could also prevent myocardial cells apoptosis probably by increasing the mRNA and protein expressions of SIRT1 and inhibiting the mRNA and protein expressions of P53, NF-*κ*B, FoxO1, FoxO3, and FoxO4 [61]; in addition, it could promote angiogenesis and inhibit vascular remodeling moderately, the mechanism of which involved multiple pathways [62]. These research results also provided important data for demonstrating the potential mechanism of XFZYD for treatment of CHD.

Potential limitations of the included trials were related to the inconsistency and variability across eligibility criteria in the original trials, as well as the variability in study design, study type, sample size, and the inconsistency in methods of measurement used across trials. For example, most of the trials were conducted in China, and some of them did not include placebo control. In order to avoid the impact on the results, this study adopts more stringent methods of literature screening and data extraction. Although it may not completely avoid the impact of risk, the effect trend of the research results can be used as a strong reference evidence.

## 5. Conclusion

In summary, multiple outcomes were used to systematically evaluate the efficacy and safety of XFZYD for CHD in this study. XFZYD could treat CHD through a comprehensive action of many herbs, which showed excellent efficacy with no obvious adverse reactions. And according to the results, XFZYD is more suitable for CHD patients with clinical indicators of dyslipidemia, high blood viscosity, or vascular endothelial dysfunction. Of course, more long-term, randomized, double-blind and multicenter clinical trials are anticipated to provide stronger evidence for XFZYD in the treatment of CHD in future.

## Figures and Tables

**Table 1:** Characteristic of the 30 trials included in the meta-analysis.

Author(s)	Country and setting	Sample size (experimental/control)	Patient enrollment time	Experimental	Control	Duration	Outcome measures
An	Liaohe Oilfield General Hospital, Liaoning Province, China	36/36	2017.1–2018.1	XFZYD + isosorbide mononitrate	Isosorbide mononitrate	1 month	Clinical effect; TCM syndrome score

Chen et al.	Beijing Daxing District People's Hospital, Beijing, China	75/75	2009.1–2011.6	XFZYD + conventional therapy	Conventional therapy	1 month	Clinical effect; electrocardiogram effect; TC; TG; LDL-C; HDL-C

Dong	Bei'an First People's Hospital, Heilongjiang Province, China	40/40	2014.5–2015.6	XFZYD + conventional therapy	Conventional therapy	3 months	TC; LDL-C

Gao et al.	Harbin First Hospital, Heilongjiang Province, China	38/38	2017.3–2018.3	XFZYD + atorvastatin; aspirin; isosorbide mononitrate	Atorvastatin; aspirin; isosorbide mononitrate	1 month	Clinical effect; NO; ET

Huang	General Hospital of Shenyang Military Region, Liaoning Province, China	20/20	2016.1–2018.1	XFZYD + rosuvastatin	Rosuvastatin	1 month	Clinical effect; TC; TG; LDL-C; HDL-C

Kong and Liu	ShunDe Hospital Guangzhou University of Chinese Medicine, Guangdong Province, China	32/32	2017.1–2018.9	XFZYD + warfarin; atorvastatin; metoprolol; aspirin; or clopidogrel	Warfarin; atorvastatin; metoprolol; aspirin; or clopidogrel	2 months	Clinical effect

Li	Shaodong County Hospital of Traditional Chinese Medicine, Hunan Province, China	45/45	2008.1–2010.1	XFZYD + aspirin; isosorbide mononitrate; simvastatin	Aspirin; isosorbide mononitrate; simvastatin	1 month	Electrocardiogram effect; AF

Li	Inner Mongolia Zhalantun city Hospital, Neimenggu Province, China	126/125	2010.6–2012.6	XFZYD + conventional therapy	Conventional therapy	3 months	AF; DAP

Li	Maoming Hospital of Traditional Chinese Medicine, Guangdong Province, China	30/30	2016.6–2017.6	XFZYD + conventional therapy	Conventional therapy	14 days	Adverse reaction

Li and Yu	Yueyang Hospital of Traditional Chinese Medicine, Hunan Province, China	30/30	2015.12–2016.2	XFZYD	Aspirin; isosorbide mononitrate; atorvastatin	2 weeks	Clinical effect; adverse reaction

Liang and Li	Central People's Hospital of Tengzhou City, Shandong Province, China	150/150	2016.10–2018.6	XFZYD + aspirin; isosorbide mononitrate	Aspirin; isosorbide mononitrate	1 month	Clinical effect; AF; DAP; adverse reaction

Liao and Liang	Maoming Hospital of Traditional Chinese Medicine, Guangdong Province, China	40/40	1999.5–1999.9	XFZYD	Isosorbide nitrate	1 month	Clinical effect; electrocardiogram effect; TC; TG; LDL-C; HDL-C; adverse reaction

Liu et al.	Chongqing Jiangjin District Hospital of Traditional Chinese Medicine, Chongqing, China	60/60	NR	XFZYD	Simvastatin	1 month	Clinical effect; TC; TG; LDL-C; HDL-C; adverse reaction

Liu et al.	The First Affiliated Hospital of Heilongjiang University of Chinese Medicine, Heilongjiang Province, China	34/34	2015.1–2016.1	XFZYD + conventional therapy	Conventional therapy	1 month	Clinical effect; TCM syndrome score

Wang	Nangong Ji Nan Great Wall Hospital, Hebei Province, China	126/125	2010.6–2012.6	XFZYD + conventional therapy	Conventional therapy	3 months	AF; DAP

Wang	The First Affiliated Hospital of Henan University of Science and Technology, Henan Province, China	50/50	2013.1–2014.1	XFZYD + conventional therapy	Conventional therapy	3 months	Clinical effect

Wang et al.	Dagang Hospital of Traditional Chinese Medicine, Binhai New Area, Tianjin, China	30/30	2012.2–2013.7	XFZYD + conventional therapy	Conventional therapy	2 months	AF; DAP

Wang et al.	TeDa Cardiovascular Hospital and Tianjin Chest Hospital, Tianjin, China	75/75	2011.1–2012.12	XFZYD + aspirin; clopidogrel; atorvastatin; metoprolol	Aspirin; clopidogrel; atorvastatin; metoprolol	1 year	Clinical effect; NO; ET

Wang et al.	Dongzhimen Hospital, Beijing, China	34/27	NR	XFZYD + conventional therapy	Conventional therapy	1 month	Clinical effect; electrocardiogram effect

Xi and Li	The First Hospital of Weinan City and Xi'an Yanliang District Hospital of Traditional Chinese Medicine, Shaanxi Province, China	20/20	2018.9–2019.6	XFZYD + metoprolol	Metoprolol	1 month	Clinical effect

Yang and Zhang	Yicheng Central Health Center in Zaozhuang City, Shandong Province, China	75/75	2015.3–2017.3	XFZYD + conventional therapy	Conventional therapy	1 month	Clinical effect

Yang et al.	Hebei Tangshan Workers' Hospital, Hebei Province, China	48/48	2013.12–2014.12	XFZYD + aspirin; isosorbide mononitrate	Aspirin; isosorbide mononitrate	1 month	Clinical effect; AF; DAP; TC; TG; LDL-C; HDL-C; adverse reaction

Yuan	Guangzhou Hospital of Traditional Chinese Medicine, Guangdong Province, China	30/30	2015.3–2016.3	XFZYD + conventional therapy	Conventional therapy	14 days	Clinical effect; TCM syndrome score

Zhang	The First Affiliated Hospital of Guangzhou University of Chinese Medicine, Guangdong Province, China	30/30	NR	XFZYD	Aspirin; atorvastatin; perindopril; metoprolol;	3 weeks	Clinical effect; AF; DAP; TCM syndrome score

Zhang	Zhengzhou People's Hospital, Henan Province, China	50/50	2008.1–2011.12	XFZYD + aspirin; isosorbide mononitrate; metoprolol	Aspirin; isosorbide mononitrate; metoprolol	1 month	Clinical effect; electrocardiogram effect; TCM syndrome score; TC; LDL-C; HDL-C;

Zhang	Traditional Chinese Medicine of Ruzhou City, Henan Province, China	52/52	2014.1–2016.1	XFZYD + conventional therapy	Conventional therapy	2 months	Clinical effect; AF; DAP; TC; HDL-C

Zhang	Songzi Hospital of Traditional Chinese Medicine, Hubei Province, China	73/73	2017.12–2019.2	XFZYD + aspirin; isosorbide nitrate	Aspirin; isosorbide nitrate	28 days	TCM syndrome score; WBV; PV; FB

Zhang and Wang	Minquan County Hospital of Traditional Chinese Medicine and The First Affiliated Hospital of Henan College of Traditional Chinese Medicine, Henan Province, China	20/20	NR	XFZYD + aspirin; low molecular heparin; nitroglycerin; metoprolol; valsartan; nifedipine	Aspirin; low molecular heparin; nitroglycerin; metoprolol; valsartan; nifedipine	1 month	Clinical effect; electrocardiogram effect

Zhang et al.	Luyi County People's Hospital, Henan Province, China	55/50	2015.9–2018.9	XFZYD + aspirin; isosorbide mononitrate; metoprolol; nitroglycerin	Aspirin; isosorbide mononitrate; metoprolol; nitroglycerin	14 days	Clinical effect; electrocardiogram effect; AF; DAP; WBV; PV; FB

Zheng	Nanping Second Hospital, Fujian Province, China	46/46	2016.1–2017.10	XFZYD + atorvastatin	Atorvastatin	1 month	AF; DAP; TC; TG; ldl-c; hdl-c; WBV; PV; FB

AF: angina frequency; DAP: duration of angina pectoris; ET: endothelin; FB: fibrinogen; HDL-C: high-density lipoprotein cholesterol; LDL-C: low-density lipoprotein cholesterol; NR: not reported; PV: plasma viscosity; TC: total cholesterol; TG: triglyceride; WBV: whole blood viscosity; XFZYD: *Xue-Fu-Zhu-*Yu decoction.

## Data Availability

The data used to support the findings of this study are available from the corresponding author upon request.

## Abbreviations

AF: Angina frequency
CD: Chemical drugs
CHD: Coronary heart disease
CI: Confidence interval
DAP: Duration of angina pectoris
ET: Endothelin
FB: Fibrinogen
HDL-C: High-density lipoprotein cholesterol
LDL-C: Low-density lipoprotein cholesterol
MD: Mean difference
PV: Plasma viscosity
RR: Relative risk
TC: Total cholesterol
TCM: Traditional Chinese medicine
TG: Triglyceride
WBV: Whole blood viscosity
XCWC: *Xue-Fu-Zhu-Yu* decoction combined with chemical drug
XFZYD: *Xue-Fu-Zhu-Yu* decoction.
